# 4468 Evaluating the Emerging Investigators Website as an Educational Resource for Early Career Researchers

**DOI:** 10.1017/cts.2020.211

**Published:** 2020-07-29

**Authors:** Layla Fattah, Inga Peter, Jenny Lin, Janice Lynn Gabrilove

**Affiliations:** 1 Mount Sinai School of Medicine

## Abstract

OBJECTIVES/GOALS: The aim of this project is to assess the usability and acceptance of a web-based educational resource for early career researchers. The Emerging Investigators website is designed to bring together resources, provide educational support and foster a community of early career researchers throughout the Mount Sinai Health System (MSHS). Locally designed and built, this web-based platform is developed using the principles of Community of Inquiry (COI), which considers how the design of online learning environments might best create and sustain a sense of community among learners. Developing a resource that meets the needs of this cohort of researchers requires an iterative implementation strategy guided by user feedback. A formal website roll-out strategy and accompanied evaluation aims to determine the design, navigability, content, relevance and educational value of this online resource from a user perspective. METHODS/STUDY POPULATION: In order to ensure this resource effectively meets the needs of this cohort of researchers, a mixed process of evaluation and design was utilized. An initial phase 1 survey was conducted with TL1 and KL2 scholars. Surveys consisted of standardized questions with answers arranged as Likert-type scales and additional written responses to collect valuable qualitative data. A convenience sample of early career researchers at Mount Sinai were contacted for initial survey participation (N = 10). A total of 3 junior faculty KL2 scholars, 3 TL1 post-doc and 4 TL1 pre-doc scholars responded to the survey. Participants were initially asked to comment on design, functionality and usefulness of content on a Likert scale with qualitative comments to support the given scores. They were subsequently asked to consider what key topics or resources were missing from the website. Based on the initial survey, changes were made to the format and content of the Emerging Investigators website to improve content relevance and usability. For phase 2, an evaluation rubric was developed to assess design, navigability, content, relevance, along with three key COI criteria to determine the educational value of this online resource. The rubric will be utilized to collect feedback in the wider phase 2 roll out of the website. RESULTS/ANTICIPATED RESULTS: The first phase of survey feedback shaped overall design of the resource. The second phase will comprehensively evaluate the value of the website in the context of teaching and learning for emerging investigators. Ten surveys were captured in the first phase. Data collection is ongoing for the second phase. Phase 1 feedback was primarily qualitative, and valuable in informing overall design choices and content. Overall the website was well received, with participants commenting on the value of the resource in terms of content and educational value. Participants particularly appreciated the regularly updated calendar function and the links provided to a wide range of resources. Functionality issues, such as broken links, were reported by participants and repaired for phase 2. Further topics of content were identified, and additional links and multimedia resources were added to address this feedback. The second phase evaluation is ongoing with data collection being conducted via an evaluation rubric. DISCUSSION/SIGNIFICANCE OF IMPACT: The Emerging Investigators website, developed using the principles of COI provides key learning, reading and resources for early career investigators in a format that is well received by a sample group of early career researchers at Mount Sinai. The website has aimed to address the reported need for communication, collaboration and social interaction with peers and other researchers across the MSHS through the addition of further web-based resources such as a LinkedIn page, a blog to feature research and provide a sounding board for research efforts, and a calendar of events targeted specifically at early career researchers. These were highlighted as areas of particular value by the participants. We anticipate the results of phase 2 rubric-based evaluations will provide actionable data that will lead to further refinement of the website, an optimized interface, and improved usability.

